# Carbon stocks of homestead forests have a mitigation potential to climate change in Bangladesh

**DOI:** 10.1038/s41598-021-88775-7

**Published:** 2021-04-29

**Authors:** Tarit Kumar Baul, Tajkera Akhter Peuly, Rajasree Nandi, Lars Holger Schmidt, Shyamal Karmakar

**Affiliations:** 1 Institute of Forestry and Environmental Sciences, University of Chittagong, Chittagong, 4331 Bangladesh; 2 Department of Geoscience and Natural Resource Management, University of Copenhagen, Rolighedsvej 23, 1958 Frederiksberg C, Denmark

**Keywords:** Climate sciences, Ecology, Environmental sciences

## Abstract

A total of 176 homestead forests at three altitudes in the Chittagong Hill Tracts, Bangladesh were randomly surveyed to estimate carbon (C) stocks and how stand structure affects the biomass C. All woody vegetations were measured, and litter and soil (0–30 cm depth) were sampled. The tree biomass C stock in the top two altitude forests was up to 37–48% higher than in low altitude, owing to significantly higher tree density and species diversity. An increase in species diversity index by one unit increased the biomass stock by 23 Mg C ha^−1^. The C stock of litterfall in low altitude forests was 22–28% higher than in the top two altitude due to the deposition of litters downslope and deliberate use of mulch for soil improvement and conservation, resulting in up to 5% higher total soil C. The topsoil C was 10–25% higher than the deeper soil, depending on the altitude. The forest stored 89 Mg C ha^−1^, indicating a potential for C sequestration in trees outside forest. This study would help policymakers to strengthen the recognition of small-scale forests for mitigation in REDD + (reducing emissions from deforestation and forest degradation, the role of conservation, sustainable management of forests, and enhancement of forest carbon stocks) and support owners through C credits from sustainably managed forests.

## Introduction

In Bangladesh, CO_2_ emissions increased by 609% from 1990 to 2017 due to increased energy consumption and land use change^1^. A gradual increase in the emissions of carbon dioxide (CO_2_) is believed to be a major contributor to global warming^2–4^. Although Bangladesh makes up only a small contribution to global emissions, the country is expected to be among the most vulnerable to climate change^5,6^. Tropical forests have the potentials of removing atmospheric CO_2_ emissions^7–9^ and store up to one-fourth of the global terrestrial carbon (C)^10^. Tropical homestead forests, are rich in biodiversity, sequester, and store C in biomass and soil^11–14^. Management strategies also affect C sequestered in homestead forests^15^. Assessing the C sequestration potential of homestead forests is essential to understand their mitigation potentials against climate change. Nevertheless, the potential of homestead forests in sequestering C has not yet been fully recognized and researched^13,16^ due to their diverse nature and thus the difficulty in the assessment.

In Bangladesh, homestead forests are some of the most productive systems and unique land uses^17^. About 80–90% of the demand for timber and fuelwood is met from the homestead forests^18,19^. Homestead forests cover 0.27 million hectares, which makes up 10% of the total tree-based land cover and 2% of the total land area^20,21^. Homestead forests thus possess the potential to sequestrate a considerable quantity of C^22–24^. However, the structural variation in a forest (e.g., tree height, diameter at breast height DBH, density, basal area BA, and species diversity) affects the C dynamics^23,25,26^.

In addition to above-ground-biomass (AGB), litter also contribute to soil organic carbon (SOC). Litter includes leaves, fine roots, and woody debris of diameter 2–5 cm^27^. The litterfalls store a small fraction of C to the AGB in forest ecosystems^28^, depending on the amount of litterfalls, forest type, and tree species^29,30^. Studies on the estimation of litterfalls C in tropical forests have been sporadic^31^. However, as it is an important C pool^32^, it needs to be taken into account when estimating the entire C dynamics of homestead forests^33^. Litters of homestead forests have traditionally been managed to form mulch and manure for reducing evaporation, conserving soil, and maintaining fertilty^31,34,35^. Moreover, litter is one of the traditional energy sources in rural households of Bangladesh^36^.

Forest soils represent a significant amount of Earth`s terrestrial C^37^. The concentration and stock of C are affected by using land for either forest or agriculture in the tropics. Previous studies found variation in soil organic carbon (SOC) in forested and degraded sites^38–40^ and in tropical agroforestry or home garden systems^41–44^, depending on AGB, tree density, and species richness. For example, tree species diversity and litter input affect SOC potential, which may also depend on other environmental factors e.g. availability of moisture^34,45^. With the slow decomposition of litter, organic matter accumulates and SOC increases, but increased water and nutrient enhance decomposition of litter and thereby decrease SOC stock under relatively dry conditions while releasing CO_2_ to the atmosphere^46,47^. Management of home garden in the form of tillage, mulching, and soil compaction may be another driving force for C exchange^48–50^. The variation in SOC stock is influenced by addition and removal of biomass due to management activities, an underlying processes in the soil^51,52^.

Under such backdrops, our study aims to estimate the carbon stocks in the hill homestead forest ecosystem (trees, litterfall, and soil) as mitigation potential to climate change. We hypothesized that this homestead forest will show similar soil C status compared to neighbouring secondary hill forests and a differential potential of C stock in variable altitude position in the hill. Moreover, homestead forests of variable altitude position may have a difference in stand structure and species diversity, which would affect the litter deposition and C stocks. Hence our second hypothesis was that tree species diversity and stand structure affect the tree biomass C stocks and as well as soil C stocks in the homestead forests. This research is a pilot study of carbon in one of the most common anthropogenic environments in Bangladesh.

## Results

### Carbon stocks of tree biomass (above ground and living roots) and fallen litter in homestead forests

The tree biomass C stock was higher in the high and medium altitude than in the low altitude homestead forests, though the difference among the altitudes was not significant (p ≤ 0.05) (Table 1). The C in fallen litter biomass decreased with increasing altitude (Table 1).

**Table 1:** Carbon stocks (Mg C ha^−1^) of tree biomass (above ground and living roots) and litterfall in the homestead forests sampled across three altitudes. ± represents the standard error of the mean. Same alphabet in different rows indicates the insignificant difference among the different altitude homestead forests (p ≤ 0.05).

Homestead forests	Tree biomass C stock (Mg C ha^−1^)	C stock of litterfall (Mg C ha^−1^)
Low altitude	28.69 ± 3.56^a^	0.04 ± 0.01^a^
Medium altitude	39.34 ± 4.87^a^	0.03 ± 0.01^a^
High altitude	42.50 ± 6.43^a^	0.03 ± 0.00^a^
Mean	36.35 ± 2.88	0.03 ± 0.01

### Stand structure of homestead forests

We sampled a total of 2873 individuals of a total of 71 tree species in the homestead forests across the three altitudes. A total of 968, 981, and 924 individuals belonging to 64, 63, and 64 species were recorded in the low, medium, and high-altitude ranges, respectively, within an average homestead forest area of only 0.04 ha.

**Figure 1:**
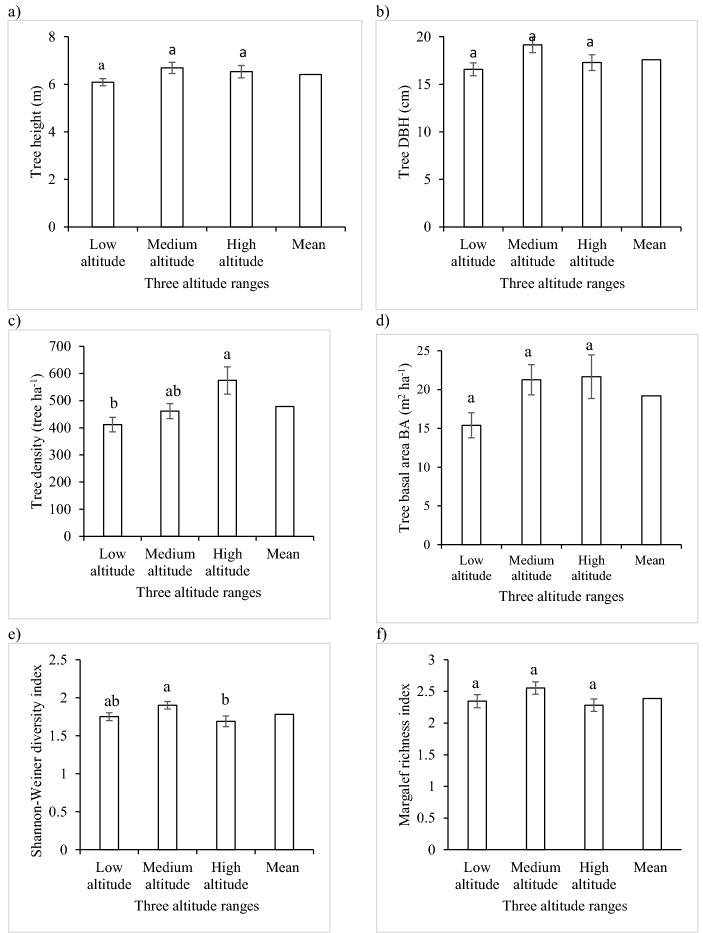
(**a**) Mean tree height, (**b**) DBH, (**c**) density, (**d**) BA, (**e**) species diversity, and (**f**) richness indices in the homestead forests across three altitudes. Bars represent the standard error of the mean. Different alphabets (a, b, and c) in the bars indicate the significant difference among the different altitude forests (p ≤ 0.05).

Mean tree height 6.1 m, DBH 17.6 cm, density 478.9 trees ha^−1^, BA 19.2 m^2^ ha^−1^, species diversity 1.8, and richness 2.39 in the homestead forests. Mean tree height, DBH, BA, and species richness in homestead forests did not vary significantly (p ≤ 0.05) among the altitudes (Fig. 1). The tree density of homestead forests was significantly (p ≤ 0.05) higher in the high altitude than in the low altitude, while in the medium altitude it was not significantly different from that in the other two altitudes. The tree species diversity of homestead forests was significantly (p ≤ 0.05) higher in the medium-altitude range than that in the high altitude, while in the low altitude it was not significantly different from the other two altitudes (Fig. 1).

### Stand structure influencing tree biomass carbon stock in homestead forests

**Figure 2:**
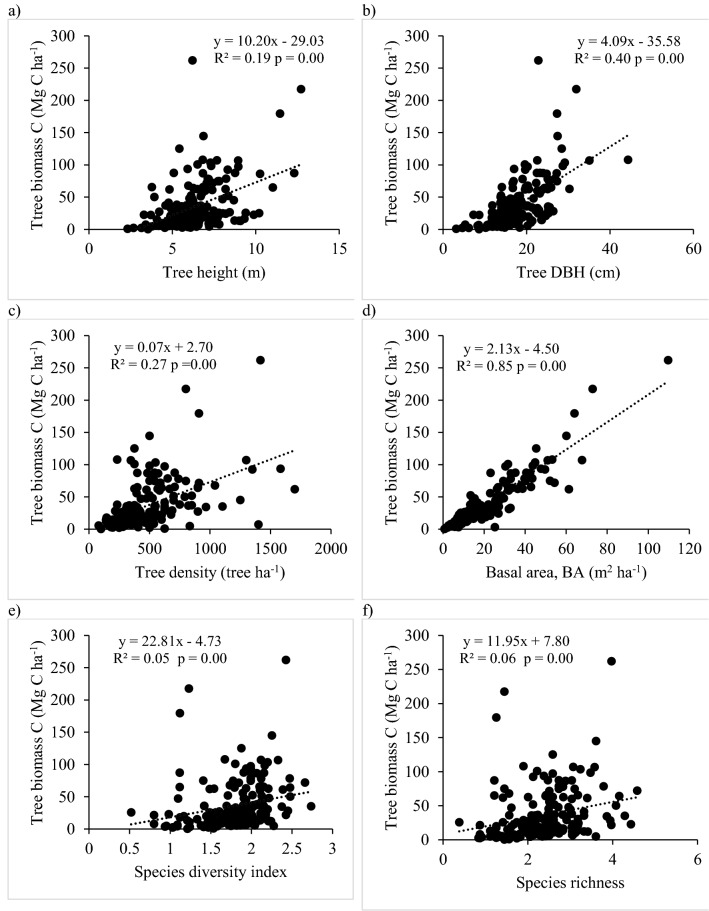
Relationship of tree biomass carbon (C) stocks with (**a**) tree height, (**b**) DBH, (**c**) density, (**d**) BA, (**e**) species diversity, and (**f**) richness in the homestead forests across three altitudes.

Figure 2 shows the significant (p ≤ 0.05) positive association between the tree biomass C and the stand structure of homestead forests. Multiple regression analysis depicted that 88% of the variability in biomass C stock was explained by the factors including the tree height, DBH, density, BA, species diversity, and richness together (Table A.1). Specifically, BA explained 85% of the total variation in C stocks (Fig. 2).

### Bulk density (BD) and soil organic carbon (SOC) concentrations and stocks in the soil

**Figure 3:**
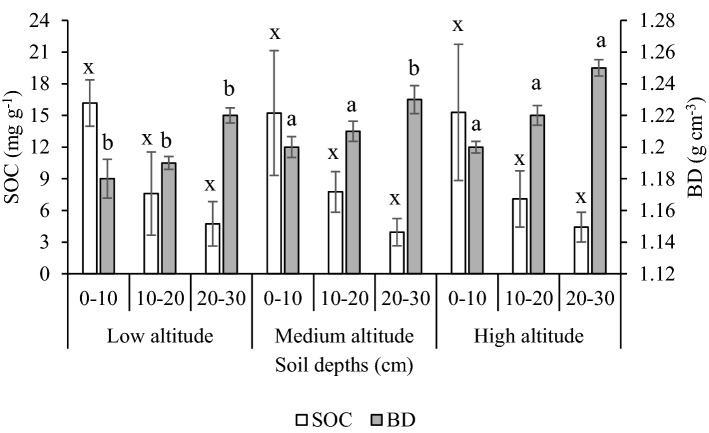
Soil organic carbon (SOC) concentrations and bulk density (BD) at 0–30 cm soil depth of homestead forests sampled across three altitudes. The secondary y-axis represents BD. Bars represent the standard error of the mean. Different alphabets (x, y, and z) and (a, b, and c) in the bars indicate the significant differences among different altitudes at the same soil depth for SOC and BD, respectively (p ≤ 0.05).

In the homestead forests, mean values in soil BD increased with depth while C concentration decreased (Fig. 3). Regarding the range, the highest BD and SOC concentration across the soil depths were in the high and low altitude, respectively (Fig. 3).

**Figure 4:**
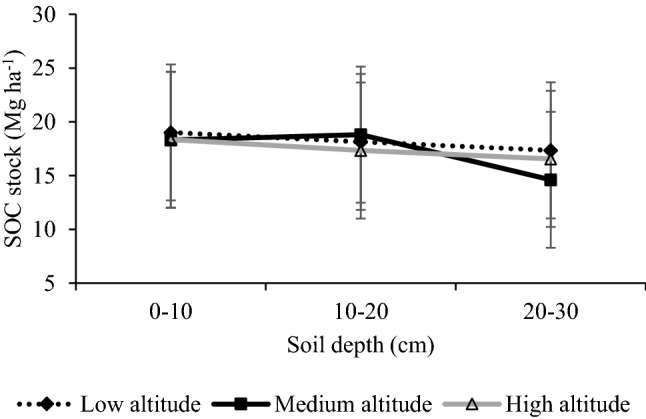
Stocks of SOC in the soil at 0–30 cm soil depth of homestead forests across three altitudes. Bars represent the standard error of the mean.

The SOC stock decreased in the homestead forests with depth (Fig. 4). Regarding the range, the highest total SOC stocks across the depths were at the low altitude forests (Fig. 4).

## Discussion

In this study, the highest tree biomass (above ground and living roots) C stock was found in the top two altitude homestead forests, which was up to 37–48% higher than at low altitude. This may be ascribed to the higher tree density, species diversity, and richness in top two altitude forests. An increase in species diversity and richness, each by one unit increased the tree biomass C stock by 23 and 12 Mg C ha^−1^, respectively (Fig. 2e,f). Our findings corroborate with other studies on homestead forests in Bangladesh and tropical forests of Asia and Africa, in which, higher tree biomass C was related to higher species diversity, richness, and tree density^23,26,40,53,54^. However, an insignificant variation in tree DBH and BA might have resulted in the insignificant differences in C stocks of biomass among three altitude homestead forests.

The estimated average tree biomass carbon (36.35 Mg C ha^−1^) in this homestead forest may be compared to Kumar^12^ estimating 16–36 Mg C ha^−1^ in Kerala home gardens in India. Conversely, these findings were only about 1/3 of those reported from the hill and mangrove forests of Bangladesh^55,56^, where the natural forests have a higher tree density, BA, and DBH. For example, compared to the findings of this study, a higher tree DBH of 10–56 cm contributed 84% of the total biomass C in mangrove forest^56^ and higher tree density (4258 ha^−1^) and BA (52.6 m^2^ ha^−1^) resulted in higher C stocks in roadside plantation^55^. Our study also revealed a significantly strong positive relationship between tree biomass C stock and BA and DBH. An increase in BA and DBH by 1 m^2^ and 1 cm, respectively increased the biomass C stock by 2 and 4 Mg C ha^−1^ (Fig. 2b,d). This was because homestead forest owners tended to reduce the tree size (height and BA) due to their small fields. Moreover, the thinning of trees and bamboo to meet the demand of timber for their consumption and household income was a regular practice, decreasing tree BA.

We found the highest C stock of litterfall in low altitude homestead forests, which was 22–28% higher than at the other two altitudes due to the deposition of litters. This may be explained by the fact that the homesteads are located on the sloping ground, where trees deposit litterfalls downslope by gravity. Litter in the low altitude had a deliberate function of pruned materials being used as mulch for erosion mitigation. Regardless of the altitude, the average C stock in litterfall was about 1% of the total tree biomass C, which was approximately half of the reported 1.8% in the hill forests of Bangladesh^29^. While litter accumulates in the natural hill forests, homestead forests, which were traditionally well managed providing branches e.g. for fuel, had much lower litter accumulation. However, litter C was not addressed as much as required in assessing ecosystem C stocks, specifically in tropical forests due to a small fraction of AGB^33^.

The bulk density (BD) of this soil (1.18–1.25 g cm^−3^) is within the range (1.22–1.58 g cm^−3^) observed in the forested and degraded area in the Chittagong hill tract^38^. The BD increased due to a reduction in litterfall deposition on the soil surface. This has also occurred in our case, a relatively smaller amount of litter and organic matter increased BD at high altitude homestead forests and in soils of the middle and deepest layers. This is consistent with Asok and Sobha^57^ indicating BD increased with depth. Périé and Ouimet^58^ found a close relation between BD and organic matter. The lower BD of the topmost layer in our samples may thus be ascribed to the accumulation of litters^59^.

We found an apparent vertical decline in concentration and stocks of SOC in three homestead forest ranges. The total SOC stock was highest (54.5 Mg ha^−1^) at the low altitude forests and the topsoil C was 10–25% higher than the deeper soil, depending on the altitude, due to the deposition of litterfall. Litter addition may enhance decomposition and trapping SOC in forests^60–62^. The overall SOC stock (52.83 Mg ha^−1^) in our study site is in line with the hill (50.5–57.6 Mg ha^−1^) and sal (58.5 Mg ha^−1^) forests of Bangladesh^21,29,62–64^, homestead forests (61.6 Mg ha^−1^), and woodlot agroforestry (48.6 Mg ha^−1^) in Ethiopia^65^. The resulted mineralization and underlying processes may influence the stabilization of C in tropical soil^51,66^. Since leaves, twigs, and branches store a significant proportion of nutrients, their removal causes a reduction in the supply of nutrients to the soil, which may lead to diminished growth. This lower growth of trees, in turn, is likely to reduce C sequestration potential and litter input in the soil. However, a long-run experimental study to observe the growth after removal of litter, branches, and performing thinning would be necessary to establish the link between litter and growth.

## Policy implications and concluding remarks

The relatively high-altitude homestead forests with higher tree density, BA, and species diversity stored 37–48% higher tree biomass (above ground and living roots) C compared to the low altitude. However, low altitude homestead forests stored up to 5% higher total SOC compared to the relatively high altitude forests due to higher deposition of litter and management of trees and litters in soil conservation. The C stock of litterfall was highest in low altitude homestead forests. The homestead forest ecosystems stored a total of 89 Mg C ha^−1^, which was higher than degraded natural forests (at 10 cm depth of soil)^40^, indicating a significant reservoir of C in the trees outside forest (TOF). The integration of indigenous management into scientific management of homestead forests can augment the potentials of C sequestration in TOF while conserving floral biodiversity, moisture, and hill soil from erosion.

Upscaling the amount of C stock to be 24 Mt for the total area of the homestead forests of Bangladesh, would have a great potential for climate change mitigation through using REDD + and CDM mechanisms. Total annual emissions in Bangladesh are 78 Mt of CO_2_^1^. The applicability of REDD + in C financing for the conservation of forests is wider, especially in tropical forests^67^, but the potentials of small scale forests such as homestead forests are ignored due to a lack of documentation of estimated C. The present documentation may help policymakers strengthening their recognition for mitigation in REDD+ and thereby support the livelihood of small-scale forest owners through ensuring sustaianble conservation and management of forest.

## Materials and methods

### Study site and sampling strategy

**Figure 5:**
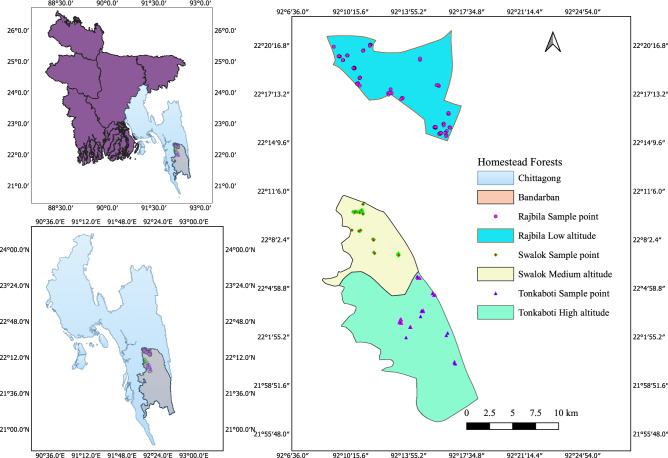
Map of the study area with sampling points of homestead forests. The Maps are created using the Free and Open Source QGIS 3.1 0, http://www.qgis.org.

We conducted this study in the homestead forests of Bandarban Sadar Upazila (sub-district) under Bandarban district located in the Chittagong Hill Tracts, Bangladesh (Fig. 5). Bandarban is a very remote and least populous district at 526–1003 masl covering an area of 4479 km^2^^68^. It enjoys a tropical climate, with a mean annual rainfall of 2630 mm and a temperature of 28 °C^69^. It comprises public forests of 322,753 ha, which are managed by the Bangladesh Forest Department (BFD) and district administration^20^. Homestead forests are owned and managed by the households. The landscape consists of steep mountains with 90% of the texture ranging from sandy loamto clay loam soil^70^. The soil is erosion-prone during rainfall; especially in connection with shifting cultivation, which is a prevalent practice. Amongst the top 10 vulnerable districts in Bangladesh, Bandarban is rated as the second most vulnerable on direct and indirect impacts of climate change^5^. The district is added as a new hotspot and will likely be the worst affected region by 2050 in terms of deforestation which has recently brought in major landslides and destruction of properties^5^.

Bandarban Sadar Upazila is situated between 21°55′ and 22°2′ N latitude and 92°08′ and 92°20′ E longitude^68^. It has a total population of around 70,000 and occupies an area of 502 km^2^, of which 85 km^2^ is forest^68^. The land is classified as a high, medium-high, low hill, and valley and high land, where dominant landuses are forest, agriculture, and shifting cultivation^71^. The local inhabitants are predominantly dependent on local natural resources for their livelihoods. Biodiversity is high but threatened by overexploitation of the resources.

Three Unions out of five selected for sampling were Rajbila, Swalak, and Tankabati, the areas of which were 10,360, 7511, and 15,281 ha, respectively^72^. We categorized Rajbila, Swalak, and Tankabati Unions as low, medium, and high ranges, respectively (Fig. 5), as they are located in the altitude of 28–43, 134, and 410 masl^72^. We assumed that every household owns a homestead forest of variable size. A total of 176 homestead forests proportionately comprising 56, 54, and 66 in low, medium, and high altitudes (Fig. 5), respectively, were randomly surveyed at a sampling intensity of 5% from the lists supplied by the Bandarban Sadar Upazila office.

### Woody vegetation survey in homestead forests

Every homestead forest, based on the size and the direction from the house, was divided into 1–15 quadrats of (5 m × 5 m) size. All woody plants except seedlings were identified and measured in height (m) and DBH (cm) in 2019 (September-December). The areas of the homestead forests (ha) were measured. We identified the tree species by the local name with the assistance of the owners of the homestead forests, and in a few cases, prepared herbarium to ensure the proper identification. A rangefinder for measuring height and a diameter tape for DBH was used. The coordinates of each sampling point were recorded by GPS.

### Sampling of soil and fallen litters

Fallen litters were collected once using a metallic frame at each of 5 points of an area of 1 m^2^ (1 m × 1 m) depending on the availability for each of the three different altitudes, thus comprising a total of 15 samples. For soil sampling, a pit of 30 cm depth, under the litter layer, was dug by using a soil auger (height 40 cm), and soil samples at 0–10, 10–20, and 20–30 cm depth were collected. Hence 15 (3 depths × 5 points) samples from each altitude-forest thus making a total of 45 (15 × 3) samples collected. Following the same procedure, 45 unaltered soil samples to measure BD were collected using a core (volume 100 cm^3^) at the same three depths in each soil sampling point^73^. The litter and soil samples collection were performed in accordance to the approval of The Director, Institute of Forestry and Environmental Sciences, University of Chittagong, Bangladesh.

### Data analyses

#### Estimation of tree biomass

Above-ground biomass (AGB) was estimated by using allometric equations for tropical trees, *Cocos nucifera*, *Areca catechu*, and *Phoenix dactylifera* (Eqs. 1–4; Table 2). Living root biomass was estimated as 15% of AGB^75^. Both AGB and living roots were summed up to estimate tree biomass and 50% C of dry mass was used to quantify total C stock (Mg ha^−1^) (Eq. 5). To estimate AGB, wood density (g cm^−3^), a required variable, was collected from Bangladesh Forest Research Institute (BFRI)^76^. For those not found in BFRI publications, we used the global wood density database^77,78^.

**Table 2:** Equations used in analyses of data.

No.	Equation	References
1	$AGB \;(kg) = (0.0673 \times ({\rho }D^{2} H)^{0.976} )$	^74^
2	*AGB *(*kg*) = 4.5 + (7.7 × *H*)	^79^
3	*AGB *(*kg*) = 10 + 6.4 H	^80^
4	*AGB *(*kg*) = − 3.956 × *H*^2^ + (55.247 × *H*) − 2.0342	^81^
5	Biomass C (Mg ha^−1^) = Biomass (dry mass, Mg ha^−1^) × 0.5 Mg C	^82^
6	$Margalef\; richness\; index = \frac{(N - 1)}{{\ln (n)}}$	^83^
7	Shannon–Wiener index, ${\text{H}} = \sum {\text{pi}} \times \ln (pi)$	^84^
8	Tree density (tree ha^−1^) = n/A	
9	Basal area, BA (m^2^ tree^−1^) = $\frac{\pi (D \times 0.01)}{4}^{2}$	^85^
10	Basal area (m^2^ ha^−1^)$= \frac{\Sigma BA}{{Area\; of \;each\; homestead \;forest \;\left( {ha} \right)}}$	^85^
11	Loss of ignition, LOI % = W1/W2 × 100	^86^
12	Soil organic carbon, SOC % = 0.47 × (% LOI – 1.87)	^86^
13	SOC stock (Mg ha^−1^) = SOC % × BD × SD	^87^
14	$Dry \;mass \;of \;litter \;sample \;(DM) \;(g) = \frac{Dry \;mass \;of \;subsample}{{Fresh\; mass \;of\; subsample}} \times Fresh \;mass \;of \;the \;sample$	^88^
15	$Litter \;dry \;mass \;per \;unit \;area\; (Mg\; ha^{ - 1} ) = \frac{DM\; (g)}{{Sampling \;frame \;area \;(cm^{2} )}} \times 100$	^88^

AGB (kg) is above-ground biomass, ρ wood density (g cm^−3^), D is tree DBH (cm), H tree height (m), N the total number of species, n the total number of individuals of all species, pi is the ratio of S to n, where, S denotes individuals of each species in a homestead forest, A an area of the homestead forest (ha), W_1_ is the loss in mass (g), W_2_ mass of oven-dried soil (g), BD bulk density of soil (g cm^−3^), SD soil depth (cm).

#### Estimation of tree density, basal area, and biodiversity indices

The tree species richness and diversity were calculated by Margalef index and Shannon-Weiner index, respectively (Eqs. 6 and 7; Table 2). The higher indices indicate higher species richness and diversity of the population. The tree density (tree ha^−1^) and BA (m^2^ ha^−1^) were also calculated (Eqs. 8–10; Table 2). Mean values of the tree height, DBH, density, BA, and all indices were compared among homestead forests of three altitudes.

#### Laboratory analysis for litterfall and mineral soil

Soil organic carbon (SOC) was determined by the loss on ignition (LOI) method following Ball. Firstly, soils were oven-dried at 105 °C for 72 h. Secondly, silica crucibles were cleaned and oven-dried by heating (at 105 °C for 30 min) and cooled in desiccators, and then weighed. Dried soils were ground by pestle and then exactly 5 g of grind soils were reweighed on an electric balance and kept in silica crucibles. The crucibles with soil were then transferred to an electric muffle furnace for igniting at 850 °C for one and half an hour. Then crucibles with soils were cooled in the desiccator and reweighed to determine LOI (%), from which, SOC (%) was calculated (Eqs. 11 and 12; Table 2). Stocks of SOC (Mg ha^−1^) were estimated using BD (g cm^−3^) (Eq. 13). To determine BD, the soil samples collected in the core segment were weighed, air-dried, and passed through a sieve (2 mm) accordingly to remove all the foreign materials, and thereafter oven-dried at 105 °C for 72 h. We calculated soil BD as the quotient between the dry mass of the fine fraction in the core segment and the volume of that soil sample.

For the estimation of C in the fallen litter, we used following method: after taking the fresh mass of the sample collected from each point, we made and labelled adequate subsamples from the weighted original sample. We then measured the wet masses of all the subsamples. Subsamples were oven-dried at 65 °C until reaching a constant mass which was recorded. Then, the dry mass of the original sample from the wet to dry ratio of the subsamples was estimated (Eqs. 14 and 15; Table 2). The C concentration was considered to be 45% of the dry mass of litter^89^ (Coleman 1972). The process was carried out for all 15 original samples collected from homestead forests across three different altitudes. C stocks (Mg C ha^−1^) in litterfalls were calculated for three different altitudes.

#### Statistical analyses

For statistical analysis, the normality of data was verified by using Kolmogorov–Smirnov (K–S) Test. One-way analysis of variance (ANOVA) determined the significant difference in mean values of tree height, DBH, density, BA, all indices, and biomass C stocks of homestead forests among three altitudes. Duncan's Multiple Range Test (DMRT) was performed to determine which homestead forest of an altitude significantly differed from the other categories of the altitudes. For mineral soil, two-way ANOVA was used to determine any statistically significant differences (p ≤ 0.05) of concentration and stock of SOC and BD against three altitude homestead forests and three soil depths. We also performed correlation and regression analyses to determine the effects of tree mean height, DBH, density, BA, species diversity, and richness on biomass C. For performing statistical analysis, we used statistical Package for Social Sciences (SPSS) 20.

## Supplementary Information

Supplementary Table.
