# Supplementary material for: Carbon stocks of homestead forests have a mitigation potential to climate change in Bangladesh
Source: Sci Rep. 2021 Apr 29;11:9254. doi: 10.1038/s41598-021-88775-7 (PMC8085129; doi:10.1038/s41598-021-88775-7)
Supplement: Supplementary file 1 — Supplementary Table. [file 41598_2021_88775_MOESM1_ESM.docx]

**Table A.1** Multiple regression analysis

| **Model Summary** | | | | |
| --- | --- | --- | --- | --- |
| Model | R | R Square | Adjusted R Square | Std. Error of the Estimate |
| 1 | .940^a^ | .884 | .880 | 13.24072 |
| a. Predictors: (Constant), Species richness, Tree height (m), Tree density (tree ha^-1^), Mean DBH (cm), Species diversity, Basal area (m^2^ ha^-1^) | | | | |
| b. Dependent Variable: Tree biomass carbon (Mg C ha^-1^) | | | | |
